# Gendered perspectives on women’s anabolic–androgenic steroid (AAS) usage practices

**DOI:** 10.1186/s12954-023-00786-x

**Published:** 2023-04-25

**Authors:** Tim Piatkowski, Jonathan Robertson, Severine Lamon, Matthew Dunn

**Affiliations:** 1 School of Applied Psychology, Griffith University, Gold Coast, Australia; 2 Deakin Business School, Deakin University, Melbourne, VIC Australia; 3 Centre for Sport Research, Deakin University, Burwood, VIC Australia; 4 Institute for Physical Activity and Nutrition (IPAN), School of Exercise and Nutrition Sciences, Deakin University, Geelong, VIC Australia; 5 School of Health and Social Development/Institute for Health Transformation, Deakin University, Geelong Waterfront Campus, Locked Bag 20000, Geelong, VIC 3220 Australia

**Keywords:** Anabolic–androgenic steroids, Harm reduction, Performance and image enhancing drugs, Steroids, Women

## Abstract

**Background:**

The masculinizing effects from anabolic–androgenic steroid (AAS) appear to be different between men and women, leading to calls for more gender-specific information regarding women and AAS use. This study sought to gather perspectives from both men and women on the unique challenges surrounding women’s use of AAS, irrespective of their personal use. Secondly, the study interrogated how women’s AAS practices differ from those of men specifically.

**Methods:**

The data presented in this paper come from a subsample of participants who participated in a larger study investigating women and performance and image enhancing drug (PIED) use in Australia. Participants were included in the current analysis if they were: (i) males or females who competed with or coached female strength athletes using AAS and (ii) female and male strength athletes who used AAS. The final sample comprised 21 participants of which there was a proportion of males (*n* = 7) and females (*n* = 7) using AAS.

**Results:**

Women’s choices in AAS selection were predominantly around oral compounds (e.g. Oxandrolone) as well as other PIEDs (e.g. Clenbuterol). Some women report the use of injectable AAS represents a change in the profile of the typical female user as it reportedly comes alongside drastic physical and psychological changes.

**Conclusions:**

The unique challenges facing women who use AAS are largely isolation and stigma, with little evidence-based practice or education being available to them online or through peer-groups. Future work may consider piloting harm reduction strategies that may be co-designed with this group.

## Background

The desire to achieve a thin physique has stemmed from women’s ideal bodies traditionally being considered ‘thin’ [1]. More recently, the ideal body type for women is changing and social influences (e.g. media) have been promoting athletic female beauty ideals alongside defined and muscular body shapes for women [2, 3]. These current body image ideals for women ascribe more muscular appearances relative to historical ideals [4, 5] and recent studies have documented a shift in the cultural ideal of physical attractiveness, with women subscribing to a visibly toned ideal [6]. Robinson et al. [6] have provided evidence for this cultural shift through women’s exposure to idealised fitness images—termed ‘fitspiration’. Specifically, participants who viewed athletic ideal images (muscular and toned) reported greater body dissatisfaction than participants who viewed traditional thin ideal images [6]. It has been suggested that the process through which this muscular ideal and body sculpting process occurs is not by female’s resisting cultural norms, instead they are hyper-conforming to them through over-identification with a hyper-idealised form of what constitutes ‘acceptable femininity [7].

To achieve this muscular body ideal men and women engage in a range of behaviours including performance and image engaging drug (PIED)/anabolic–androgenic steroid (AAS) use [8–13]. The use of PIEDs among women is not new however, as evidenced by women historically striving for a thin ideal utilising diet pills [14, 15]. Further, research has reported women also utilise other PIEDs, such as tanning agents, in an effort to modify or enhance their appearance [16]. Given the emerging body ideal of women who are seeking to achieve more muscular physiques, this group may be at risk of engaging in PIED or AAS strategies more similar to those of men. The propensity for women to engage in these strategies has been previously sequestered to women’s bodybuilding [17, 18] and, more recently, figure and bikini competitions [19]. However, overall, little attention has been given to the broader context of women regarding PIED and AAS use.

AAS are presently the most widely used PIED [20] and are comprised of testosterone, its precursors and its metabolites [21]. Exogenous AAS can be administered either orally or by intramuscular injection. The two most common therapeutical uses for AAS rely on their ability to assist in treatment of certain types of anaemia and their ability to stimulate sexual development in hypogonadal males [22]. AAS can pose various medical complications including suppression of normal neuroendocrine function, adverse effects on lipoproteins, and hepatotoxicity in the case of those orally administered [23–25].

AAS use among women is less investigated than among men. The global lifetime prevalence rate for males is 6.4%, with female prevalence rates being significantly lower at 1.6% [26]. In Brazil, Abrahin et al. [27] sampled 361 females who weight trained. Using self-report measures they found a prevalence rate of 13.3% (*n* = 48), with women primarily turning to oral compounds such as Oxandrolone and Stanozolol. The participants reported adverse effects that included acne, menstrual irregularities, water retention, voice deepening, and clitoral enlargement. Notably, some side effects may be long-lasting. Chadwick et al. [28] have shown that although some improvement may result from cessation of AAS, voice changes can be permanent. This may be due to AAS causing changes in the female neuroendocrine axis, such as reduced levels of luteinizing hormone (LH), follicle-stimulating hormone (FSH), and estrogen, in addition to increased circulating levels of testosterone (Wood, 2008). There has also been some research to suggest the distinct psychological effects of AAS use for women, including hypomania and depression [29]. More recently, AAS use among females has demonstrated further psychosocial dysfunction including poor emotional management and reduced empathy and impulse control [30]. However, compared to men, women experience lower patterns of aggression and psychological distress as a consequence of AAS use [31]. Relatively little research has been directed to explore women’s experiences of using these compounds to further elaborate on these psychosocial and gendered differences.

Qualitative work from Ip and colleagues examined a sample of female AAS users through web-surveys [32]. Their findings demonstrated that women experience AAS use differently to men and, therefore, may have their own unique needs. More recently, Havnes et al. [33] explored the experiences and harms of women who use AAS. Their study utilised a sample of women (*n* = 16) aged 18 and above who reported current or past AAS use. The authors linked AAS initiation to male partners, friends, and coaches and the participants entrusted this group with decisions about use and regarded them as reliable sources of information. The oral compounds that are reportedly often used by women, such as Oxandrolone (Anavar) and Stanozolol (Winstrol), have toxic effects on the liver, negatively affect cholesterol and lipoproteins, and increase cardiovascular risk [25]. Given the potential for health-related complications, there are some AAS users who will display help-seeking behaviours. For example, Zahnow et al. [34] have suggested that women AAS users are more likely than men AAS users to engage with a general practitioner. More recently, Havnes and colleagues have argued that few women seek information about AAS-related side effects, and that several health risks do not present symptoms, presenting a clear need to understand more about women’s AAS use [33, 35]. The potential for developing irreversible masculinizing effects from AAS is, at times, difficult for women to process and may negatively impact their well-being [33]. Specifically, the masculinizing effects that come from AAS may negatively influence self-esteem, social life, and sexual function [33], and these effects appear to be different from those male users are concerned with [36].

Recently, there has been growing interest in the practices of women who use AAS [30, 31, 33, 37]. This development is, in part, due to the increasing recognition of the distinctive challenges and risks faced by women using substances [38, 39], particularly AAS [33, 37]. This study contributes to our understanding of gender-specific experiences of, and outcomes from AAS use. There is also a growing recognition of the need for a gender-sensitive approach to harm reduction, which considers the specific needs and experiences of women from various perspectives [40, 41]. The present study contributes to knowledge by extending our present understanding of women’s use of AAS. Firstly, the study sought to gather perspectives from both men and women on the unique challenges surrounding women’s use of AAS, irrespective of their personal use. Secondly, the study interrogated how women’s AAS practices differ from those of men specifically.

## Methods

### Sampling and recruitment

Twenty-one semi-structured interviews were conducted online (via Zoom) with participants in Australia (see Table 1). The participants and data in this paper come from a larger study exploring issues related women and doping in Australia. A purposive sample of participants was recruited via researcher contacts and through snowball sampling. Interested participants were asked to contact a member of the research team; all interviews were conducted one-on-one with the first author who has significant experience in qualitative research with this population. Ethical approval was granted from the Deakin University Human Research Ethics Committee HEAG-H 135_2021.

**Table 1:** Participant characteristics and roles

Participant	Gender	Role	AAS use
1	M	Bodybuilder and powerlifter	Y
2	M	Strongman Gym owner	N
3	M	Bodybuilder and powerlifter Coach	Y
4	M	Powerlifter Coach	Y
5	M	Coach	N
6	F	Strongman	N
7	F	Strongman and powerlifter	N
8	M	Bodybuilder	Y
9	F	Powerlifter	Y
10	M	Powerlifter Coach and Gym Owner	Y
11	M	Powerlifter Coach	Y
12	F	Powerlifter Coach	Y
13	F	Powerlifter	Y
14	F	Powerlifter	Y
15	F	Powerlifter	N
16	F	Bodybuilder and powerlifter Coach	Y
17	M	Strongman Coach	Y
18	F	Strongman and powerlifter	N
19	F	Powerlifter	Y
20	F	Powerlifter	N
21	F	Strongman	Y

In line with the aim of gathering perspectives of the unique challenges facing women using AAS, there were some broader inclusion criteria. Participants were eligible to be included if they were: i) females or males who competed with or coached female strength athletes using AAS. Regarding the study’s second aim of interrogating differences in women’s practices, participants were eligible to be included if they were ii) female and male strength athletes who used AAS. These criteria were employed to give weight to the multiple attitudes, perceptions, and understandings which may contribute to extending knowledge of gendered differences in AAS use. The participants fit within a variety of roles: coach = 10, gym owner = 2, strength athlete = 19. Fourteen participants reported AAS use, and half of these were female. Of the participants using AAS, this cohort were all using testosterone or some derivative of testosterone orally and/or intramuscularly. Most participants were long-term users and had completed multiple 'cycles' (AAS combination and duration of use). Many also 'stacked' (combined) their AAS (injectable and oral) with other performance and image enhancing drugs (PIEDs) such as Clenbuterol, insulin, or peptides.

### Materials and data collection

Interviews were conducted between September and December 2021 and were between 25 and 90 min in duration. All interviews were conducted online due to the COVID-19 restrictions, digitally recorded and transcribed. A semi-structured interview schedule was used. The questions included a uniform set of ‘prompts’; some were related to gaining information about the participant and building rapport (such as their working background, years competing in strength sports), while others were related to the study and its aims, such as the unique way in which women using AAS are seen by others, and the practices they engage in. For example, questions included: Can you tell me a little bit about how AAS use plays a role in your strength sport? What is it like working with or competing against women who use AAS? Do you think there are any differences between AAS use among women and men? No reimbursements were offered for participation. The first author is active in community engagement with this group and provides ongoing harm reduction through professional networks; this practice likely creates strong network bonds, which allow for these types of methods to be employed.

### Data analysis

The first author transcribed all interviews. Once the transcribing process was complete, the finalised transcripts were checked by the first author prior to analysis. Preliminary impressions and reflections were noted following the interviews. Data were imported into NVivo (Version 12) for further analysis. Within the NVivo platform, the transcripts underwent preliminary analysis by the first author, with input from the other authors at regular research meetings. The data were analysed inductively [42] and the codes identified were then synthesised into themes to summarise the key findings from the interviews. The analyses were performed through a lens of social constructivism deemed appropriate for research in this area and giving weight to participant discourse and narratives [43]. The data were thematically analysed [44]. The analytical process followed Braun and Clarke’s six-step guide [45] which involves data familiarisation; initial code generation; identification of potential themes; review of themes; defining themes; and compilation of findings. The first author generated codes, then further organised and conceptually supported these codes by relevant examples from more than one transcript—a reflexive process where themes were developed through re-engaging with the written transcript and the recordings available [45, 46]. Further research team meetings were held to scrutinise the results of coding decisions and reflect upon themes and patterns emerging in this process. During these research meetings all Authors assisted in refining and defining themes. These meetings represent a coding approach that is collaborative and reflexive, designed to develop a richer more nuanced reading of the data, rather than seek consensus on meaning [46]. Throughout this process, a list of verbatim quotations illustrating each theme was compiled and a list of theme titles and representative extracts was created. The themes were generated from participant responses to align as closely as possible with their language (Table 2).

**Table 2:** Coding process

Data extract	Coded for	Potential theme	Overarching theme
*“I think there's like that whole like feminist movement going and stuff like I think like you know it's cool to see like I like I love it too it's like it's cool to see like strong girls and like girl supporting each other.”*	Strength sport, support, females, femininity, powerlifting	Feminine Empowerment in a Masculine Landscape	Women in strength sports—feminine empowerment
*“Like what what would what would a girl prefer to use would she prefer to inject NPP or take take anavar just take a few tablets today, of course, she's going to choose the tablets”.*	AAS use woman, inject, injectables, oral, gender differences, preference	Choosing Anavar	Perceived harm reduction: “Rather go orals than injectables”
*“As a female obviously the substances that you do use can have a lot more damage than a male using them, I mean obviously damage can be done, but as a female it can really like screw you up.”*	AAS use, gender differences, female sides	The unique consequences of AAS use for females	For Females—It’s going to come with other S**t as well!
*“I think it's typically something that was known for being used predominantly in the male world. um. So perhaps there's a bit of a stigma there um Where females are going I don't want to discuss that.”*	AAS use woman, male, gender, unseen, silence, stigma	The ‘unseen’ nature of AAS use among women	The role of stigma in women’s use of AAS

### Reflexivity statement

The first author conducted all interviews for this study and has been an active member of Australia’s strength sport communities. The author acknowledges his significant lived experience and provides professional training to organisations in Australia who engage regularly with AAS consumers. He maintains professional and personal networks with a variety of individuals involved in the alcohol and other drugs space. Throughout this process, the first author reflected regularly on his research experience. The second author is engaged in sport management and athlete well-being, considering the impacts of doping. The third author is a molecular biologist who focuses on the role of skeletal muscle mass in relation to health. The fourth author performs research with PIED users, in an effort to reduce harm and optimise health outcomes. The paper’s co-authors also reflect upon their experiences with fitness industries and communities, as well as perceptions of illicit drug users, in their approach to providing critique and feedback for data.

## Results

Twenty-one participants (12 female, 9 male) consented to be interviewed. There were four overarching themes developed from the data, see Table 3.

**Table 3:** Overarching themes and sub-themes

Overarching theme	Sub-themes
Theme 1: Women in strength sports—feminine empowerment	1. Femininity and masculine landscapes
	2. Social acceptance of muscle
	3. Strength sports and AAS
Theme 2: Perceived harm reduction**—**Rather go orals than injectables	1. Choosing Anavar
	2. The injectable shift
	3. Reducing harms
Theme 3: For females—it’s going to come with other s**t as well	1. Impact of AAS on women
	a. Physical changes
	b. Psychological changes
	2. Men’s perspectives of women’s AAS harms
Theme 4: The role of stigma in women’s use of AAS	1. Gendered secrecy
	2. Stigma and judgement
	3. Peer-education and support

### Theme 1: women in strength sports—feminine empowerment

Several of the participants were vocal about the benefits of women participating in more male-dominant sporting contexts. This participation was positive as it was perceived as a symbol of feminine empowerment by participants. Further, this growing participation tended to be linked to feminist movements. The women interviewed were especially vocal regarding the expansion of previously exclusively masculine spaces to be a place where they felt supported:

[On women’s increasing participation in strength sports]

Px12[Female Powerlifter and Coach, AAS-User]: I think it’s just um, like women doing more dominating sports in general. So not just powerlifting you're seeing boxing, MMA [mixed martial arts], BJJ [Brazilian Jiu Jitsu] like anything like that it's just growing a lot. Women feel more confident to get into these sports. which has been really cool.

Px13 [Female Powerlifter, AAS-User]: I think there's like that whole like feminist movement going and stuff like I think like you know it's cool to see like I like I love it. It's cool to see like strong girls supporting each other, and I think like that whole thing has like really like grown up and helped it by pushing it along I think.

The attitude of feminine empowerment extended to strength sports. These sports, such as powerlifting, are seen as a salient avenue where women were able to feel empowered by expressing their femininity in a (traditionally) masculine context:

Px14 [Female Powerlifter, AAS-User]: You know I really felt empowered as a woman, I think there's something to be said for people, women in strength sports, it is very empowering*.*

Participants expressed that the change in women being more involved in strength sports may also have links to other sociocultural shifts. Specifically, women in this cohort felt as though it was more socially acceptable to be perceived as strong and, therefore, potentially carry more muscle mass. These changes highlight the link between the emerging body ideal trend among females (muscular and toned) and how it is influencing traditional gender norms:

Px20 [Female Powerlifter, Non-User]: But I also think it's becoming a little bit more socially acceptable for women to be strong and to potentially carry a little more muscle than what we would traditionally see as the norm for women.

As a result of adhering to the emerging muscular body ideal for women, and through strength sport participation, some women reported heightened propensity to engage in AAS use.

Px09 [Fe Female Powerlifter, AAS-User]: I think a big contributor, now that I look back, so the contributor for me going down the road of performance enhancing drugs was getting into those higher PL [powerlifting] competitions and seeing these other big, like muscular and big women.

### Theme 2: perceived harm reduction: “rather go orals than injectables”

The participants in this study confirmed what is already well-established in strength athlete communities and academic literature: that females who use AAS tend to opt for compounds they can ingest orally:

Px01 [Male Powerlifter, AAS-User]: Yeah I think girls well females are a lot more comfortable using orals or compounds um especially things like to clenbuterol is obviously up there, I think…big ones are clenbuterol and Anavar [Oxandrolone].

Px21 [Female Strongwoman, AAS-User]: [discussing the reasons people choose not to inject] they just assume that orals are a um you know a bit more of a safer option.

Notably, these data provide some indication of the reasoning for these choices, through each participants’ respective lens of knowledge. For instance, participants who were interviewed were aware of women’s practices in this space and indicated that the use of oral compounds was preferred to injection as the practice of ‘punching in’ (injecting) an AAS made it much more ‘real’. The use of oral compounds alleviates tension and may be perceived as more socially acceptable:

Px08 [Male Bodybuilder, AAS-User]: From what I hear a lot of a lot of women, try to go for the Anavar sort of route, whereas they don’t have to pin [inject]… Because it's fine you know it's there's no harm and it's not it's not a proper steroid you’re not, you know punching it in [injecting]… And I think that’s what alleviates some of that tension from, as well as just a tablet like you know couldn't do much damage right. And yeah, so I think it takes a realness away from it, but I think when somebody is confronted with a pin [needle] it's a very different story. But yeah I think a little, women lean towards Anavar.

The women who were using AAS and opting for oral compounds expressed their concern around side effects and potential health complications that could arise as a result of use. AAS use may come with visible side effects, which some women in the present study linked to injectable use. This link likely exists, but is due to the higher dosages administrable via injection and potential for stacking both oral and injectable compounds rather than mode of administration. Participants who use only oral compounds argued that injectable AAS may bring more obvious physical side effects given their observations of the women in strength sports who have disclosed injectable use. Notably, injecting AAS tended to represent the transition across a hidden boundary between oral user and injectable user. Participants indicated that this transition to using a needle also had negative connotations associated with being a drug user, unlike taking a tablet. Therefore, the role of stigma may also play a part in participants choices around compounds selection, for instance:

Px12 [Female Powerlifter and Coach, AAS-User]: I think, just for me personally I’m never going to do anything other than orals… it's not really an interest to me.

Interviewer: Can I ask what made you have that the decision not to use injectables?

Px12 [Female Powerlifter and Coach, AAS-User]: I just don't feel like I need it. I like I’ve definitely thought about it, like numerous times but yeah definitely I’ve seen a lot more worse side effects, than good I guess. I’m around top athletes all the time and the top [Removed] in Australia they're all heavily you know heavily use PEDs [Performance Enhancing Drugs]. A lot of them have really bad acne, they have really deep voices, they’re growing hair in different places, they've got really big jawlines, and it's just me I don't get paid to do powerlifting I just don't see the worth in it.

Px19 [Female Strongwoman, Non-User]: I think the main reason that that women would rather go orals than injectables is the the stigma around it. Like Oh if I’m only taking a tablet or it's not that dangerous or it probably is going to do as much to me. But as soon as you go I’m using a needle to put a substance into my body that can have negative connotations, and then you kind of get put into that like basket of being a drug user because you're doing it with a needle.

Of the women who did choose to progress to injectable compounds, many indicated their understanding regarding how ‘extreme’ the behaviour was. The women who used injectables did not necessarily stop using oral compounds and, instead, tended to ‘stack’ more drugs of increasing variety, for example:

Px16 [Female Bodybuilder/Powerlifter and Coach, AAS-User]: [discussing competition preparation] And I’ll just give you an idea, but in my last four weeks I was to drop my Anavar. I was on 75 mils of Tren A [Trenbolone Acetate] every second day, Sus250 [Sustanon250 Testosterone Blend], 2 mils twice a week, Boldenone or Equipoise [Boldenone Undecylenate], 2 mils twice a week, Anadrol [Oxymetholone] 50 mil [milligram] tab every day. That is a lot of s**t.

### Theme 3: for females—it’s going to come with other S**t as well!

Men who had known female AAS users for a significant time were able to gain insight regarding women’s usage practices. These men were usually AAS users who competed at strength sport competitions which women who used AAS frequented and had built rapport and even friendship over a number of years. Alternatively, they were coaches who had input into women’s training and, therefore, knowing about their specific AAS regimen was of importance for exercise programming. Notably, some participants managed to fill both roles.

Px03 [Male Powerlifter and Coach, AAS-User]: Over time you get to know some of the girls like NAME, and NAME and you know, eventually it [AAS use] comes up in conversation. That takes time though right like and with like coaching you know you see them recovering quicker and whatever and you might say what’s going on here. Or they offer it [AAS information] up themselves.

Male participants in this study recognised the impact that using AAS could have on women’s general health and well-being. The consequences for women were believed to be more harmful overall, which likely plays a role in the attitudes of others toward the women who are using. Specifically, participants expressed that the acceptability of women using these types of substances was still quite low and their use brought a high level of scrutiny. For example:

Px11 [Male Powerlifter, AAS-User]: I guess if an 18 year old kid as a bloke does it uh the repercussions are nowhere near as bad as when an 18 year old girl does it.

Px17 [Male Strongman, AAS-User]: Yeah it's definitely more acceptable for men to take and it's more acceptable to be spoken about. Generally, when you hear people talk about a guy taking stuff everybody kind of brushes it off. Whereas if people are sort of gossiping and they're like I think she's taking stuff it's really put in like a negative way a lot of the time.

The women who used AAS did substantiate the extent of these potential harms. For females, the use of these substances had far-reaching consequences. The women who chose to use AAS were vocal about the physical and psychological impacts of these drugs. Below are some short participant narratives providing some insight into the lived experiences of women who use AAS:

Px09 [Female Powerlifter, AAS-User]: Who wants to shave like and that's where I guess psychologically you're like, you know when you're standing in the mirror looking at yourself and you've got facial hair that's when you start to go to yourself what have I done to myself. I’ve done this to myself.

Px16 [Female Bodybuilder/Powerlifter and Coach, AAS-User]: It’s s**t house. If girls think that you know, not taking testosterone is not going to change you well sorry it is. You don't all of a sudden just become a champion bodybuilder or lift amazing weights it's going to come with other s**t as well. And don't think that I haven’t thought about this, I think about this every day. I don't think there's anything anyone could say to me that is more painful than questioning my gender.

Px21 [Female Strongwoman, AAS-User]: I don't know if it was maybe just an internal kind of stress of me but I’ve always had quite like a deep voice like growing up… I’ve always kind of had like a bit of a husky voice, but in my head I felt like my voice was deepening and it was something that I was really concerned about.

### Theme 4: the role of stigma in women’s use of AAS

The participants in this study indicated that, although men who use AAS are reportedly secretive, there is a level of peer education and support that occurs within the male cohort. In contrast, women’s silence regarding the use of AAS brings with it several additional health-related challenges, for example:

Px02 [Male Coach, Non-user]: I have experienced firsthand that women are unwilling to talk about what they're doing. Where I personally know somebody who was taking gear [AAS] uh refused to talk about it. Had some people who were looking after her health and said hey, we just need to know what's going on and we're not being judgmental. Still refused to talk about it and a couple months later, after that had some negative health outcomes. Finally, they admitted it and said yeah I was doing that the entire time.

These challenges of gendered secrecy may be somewhat attributable to a perception of additional stigma for women who use AAS. Participants in the present study expressed that this stigma may have links to the potential for AAS to have masculinising effects:

Px15 [Female Powerlifter, Non-user]: I don't know why it's not discussed very openly amongst women um maybe because I guess typically when you (pause) I know when I think of steroid use I think it's typically something that was known for being used predominantly in the male world. So perhaps there's a bit of a stigma there um where females are going I don't want to discuss that I’m using because, and I mean realistically some of the hormones that they put in their body are those that are naturally occurring in the male body aren’t they. So perhaps that's why it's not discussed so much in the female world.

The perception of the lack of peer communication was also voiced by those women who were using AAS. Women who used AAS underscored that having other females to confide and discuss these types of unique experiences with was extremely rare. However, when given the option to discuss and share their experiences, women felt quite relieved and appreciated a space where they could be open, for example:

Px16 [Female Bodybuilder/Powerlifter and Coach, AAS-User]: No one f**king talks about it. I’ve only ever had one girl who messaged me and asked me has X, Y and Z happened to you. And then she owned that she had ingested some [steroids] and I was like oh my God, yes, you know, and it was almost like a relief to actually like confide and I think she kind of felt the same way.

## Discussion

This study sought to make a novel contribution to the literature regarding gender-specific information about, and women’s experiences of using AAS. This research extends the present knowledge available on women who use AAS in line with its two aims. Firstly, to gather perspectives from both men and women on the unique challenges surrounding women’s use of AAS, irrespective of their personal use. Secondly, to interrogate how women’s AAS practices differ from men. The data show that some women are reportedly feeling more empowered to participate in strength sports and carry more muscle mass. As a consequence of participating in these sports, there may be an increased opportunity for women to go on to use AAS, as indicated by participants. For those women who do go on to use, their choices in AAS selection are predominantly around oral compounds (e.g. Oxandrolone) as well as other PIEDs (e.g. Clenbuterol). Some women report the use of injectable AAS represents a change in the profile of the typical female user as it reportedly comes alongside drastic physical and psychological changes. Women who report injecting suggest there is also a psychological shift in the way they self-identify as an AAS user. In turn, the unique challenges facing for women who use AAS are inclusive of further isolation and stigma, with little evidence-based practice or education being available to them online or through peer-groups.

Traditional conceptualisations of women participating in sport point towards a socially acceptable construction of females having to subtly balance hegemonic femininity and athleticism [47]. For instance, in diverging from this traditional balance, female bodybuilders have been previously conceptualised as ‘gender outlaws’, accruing stigmatisation as a result of the femininity-transgression taking place as a result of the demands of aesthetic strength sport [18]. Ainsworth et al. also highlight the delicate balance between remaining feminine while participating in sports dominated by male athletes, such as strength sports [48]. In less traditional sport settings, females have an ongoing reputation of resisting and challenging expectations of hegemonic femininity [47], particularly bodybuilding [49], and the present data support this ongoing transformation. Women’s participation in strength sports and the link to feminine empowerment appears to contribute to the salience of a sporting context where they can demonstrate strength and add muscle mass. The women in this study acknowledge that it is becoming more socially acceptable to carry more muscle mass and participate in previously male dominated sporting contexts. This shift in sociocultural norms underscores the emerging muscular and toned ideal [6], which our data also support. However, participation in strength sports such as powerlifting, strongman, and bodybuilding, does appear to have ties to increased AAS use among males [50] and potentially among females [51, 52]. Further, this AAS use has also been conceptualised as a manifestation of male hegemonic patterns [53], however, our data appears to provide some nuances to these patterns which have been previously unconsidered.

The present data demonstrate women’s choice to opt for oral compounds such as Oxandrolone (Anavar), and this finding substantiates the current evidence available in this area [33]. In extending on why women choose to use these compounds, our study indicates the transition to injecting AAS is one which fewer women are inclined towards. Injecting AAS represents an invisible boundary for some women, and this had connotations with stigma of drug use. The stigma that surrounds people who inject drugs (PWIDs) [54, 55] and women who inject drugs (WWIDs) [38] has been demonstrated in intravenous (IV) drug use settings. WWID may be even more stigmatised as they are both breaking the law in terms of their illegal injecting drug use, as well as transgressing the norms of conventional femininity [39]. In drawing together extant work and our current data, we suggest that WWID, namely AAS, intramuscularly (IM) represent an even more marginalised population than IV users, particularly given the illegal characteristics and visibly extreme contravention of traditional gender norms. On a physical level, participants also expressed their observations of women who had crossed this line and the obvious changes that occurred as a result. Specifically, participants who had transitioned to injecting reported how alarming physical side effects were (e.g. daily shaving, voice deepening), which builds on previous work with women [11, 33, 48]. Some women competing in elite strength sport expressed that the lack of financial reward, for example, meant that the benefits of using injectables did not outweigh the possible downsides. Further research is needed to understand the precise motivations of those women who do go on to inject AAS, which will likely provide some insight into the stigma surrounding this unique group of PWIDs.

Among men who use AAS, research has found that a group identity emerges as a result of the common interests, which centre on training, diet, and the co-occurring substance use [8, 10, 56]. Specifically, there is a level of peer-led education and harm reduction occurring within these peer networks—a ‘safe space’ dynamic [36]. These peer networks extend into online forums [57] and may be partly due to the lack of appropriate harm reduction responses and frameworks available for this group. For example, previous research demonstrates that women searching for advice and the experiences of other women regarding AAS use must navigate male contributions on internet forums [58]. For women who use AAS, our data indicate that a harm reduction dynamic does not exist presently. Females who choose to use these drugs are more secretive, and this likely has links to increased stigma as indicated by some participants. Despite the recognised health complications, these findings are indicative of barriers to meeting women’s needs for accurate information about health risks among women who use AAS, as is evidence by research with women who use and inject substances more broadly [38, 59]. More research is required to understand how these challenges are experienced and navigated by AAS-using women to inform gendered approaches to harm reduction. Future research should attempt to explore how to integrate peer-led approaches and harm reduction frameworks more effectively among women using AAS.

### Strengths and limitations

The present study engaged with a large number of participants considering the qualitative methodology, however, we recognise that this included only a relative proportion of female (*n* = 7) and male (*n* = 7) AAS users. The variety of participants allowed this research to approach the unique practices of women using AAS from a variety of perspectives and through several lenses which included a variety of attitudes, perceptions, and experiences. The lenses included the lived experience of women who used AAS and the observations of those around them. The interviews addressed sensitive topic areas and were conducted by a researcher who has extensive expertise with this group of substance users, and the potential biases that may arise from this are rightfully acknowledged. We believe this approach remains a strength of the research, however, more methods of qualitative rigour (e.g. member checking and validation) could be incorporated in future qualitative work with women using AAS. Nonetheless, the findings cannot be generalised to all women who use AAS, and further research is required to explore the motivations of women to use these substances more fully. Notably, this is an emerging research area where exploratory and foundational research should be conducted. These calls for research are in the interest of generating early and potentially pre-emptive responses among practitioners, researchers, and policy makers.

## Conclusions

This study addresses the gender-specific information gap regarding women and AAS use. The present data extend the current understanding around the gender-specific practices of women who use AAS and direct future research towards the challenges that women’s unique practices of use may bring. Further research is required to more fully understand the motivations and experiences of women who are using these substances. Coordinated harm reduction responses are necessary to provide the appropriate and targeted support for this highly stigmatised and isolated group.

## Data Availability

The data analysed during the current study are available from the corresponding author on reasonable request.

## Abbreviations

AAS: Anabolic–androgenic steroids
FSH: Follicle-stimulating hormone
IM: Intramuscular
IV: Intravenous
LH: Luteinizing hormone
PIEDs: Performance and image enhancing drugs
PWIDs: People who inject drugs
WWIDs: Women who inject drugs

## References

[1] Tiggemann M, Pickering AS (1996). Role of television in adolescent women's body dissatisfaction and drive for thinness. Int J Eat Disord.

[2] Schaefer MK, Salafia EHB (2014). The connection of teasing by parents, siblings, and peers with girls' body dissatisfaction and boys' drive for muscularity: the role of social comparison as a mediator. Eat Behav.

[3] Tiggemann M, Zaccardo M (2018). ‘Strong is the new skinny’: a content analysis of# fitspiration images on Instagram. J Health Psychol.

[4] McCreary DR, Sasse DK (2000). An exploration of the drive for muscularity in adolescent boys and girls. J Am Coll Health.

[5] Rodgers RF, Franko DL, Lovering ME, Luk S, Pernal W, Matsumoto A (2018). Development and validation of the female muscularity scale. Sex Roles.

[6] Robinson L, Prichard I, Nikolaidis A, Drummond C, Drummond M, Tiggemann M (2017). Idealised media images: the effect of fitspiration imagery on body satisfaction and exercise behaviour. Body Image.

[7] Kotzé J, Richardson A, Antonopoulos GA (2020). Looking ‘acceptably’feminine: a single case study of a female bodybuilder’s use of steroids. Perf Enhancem Health.

[8] Piatkowski TM, White KM, Hides LM, Obst PL (2020). Australia's Adonis: understanding what motivates young men's lifestyle choices for enhancing their appearance. Aust Psychol.

[9] Piatkowski TM, White KM, Hides LM, Obst PL (2021). The impact of social media on self-evaluations of men striving for a muscular ideal. J Commun Psychol.

[10] Piatkowski TM, Dunn M, White KM, Hides LM, Obst P (2021). Exploring the harms experienced by Australian men who use performance and image enhancing drugs alongside alcohol and other drugs. Perf Enhancem Health.

[11] Börjesson A, Ekebergh M, Dahl M-L, Ekström L, Lehtihet M, Vicente V (2021). Women's experiences of using anabolic androgenic steroids. Front Sports Active Living.

[12] Börjesson A, Ekebergh M, Dahl M-L, Ekström L, Lehtihet M, Vicente V (2021). Men´ s experiences of using anabolic androgenic steroids. Int J Qual Stud Health Well Being.

[13] Börjesson A, Lehtihet M, Andersson A, Dahl ML, Vicente V, Ericsson M (2020). Studies of athlete biological passport biomarkers and clinical parameters in male and female users of anabolic androgenic steroids and other doping agents. Drug Test Anal.

[14] Frank RE, Serdula MK, Adame D (1991). Weight loss and bulimic eating behavior: changing patterns within a population of young adult women. South Med J.

[15] Khan LK, Serdula MK, Bowman BA, Williamson DF (2001). Use of prescription weight loss pills among US adults in 1996–1998. Ann Intern Med.

[16] Hibbert MP, Brett CE, Porcellato LA, Hope VD (2021). Image and performance enhancing drug use among men who have sex with men and women who have sex with women in the UK. Int J Drug Policy.

[17] Boyle L (2005). Flexing the tensions of female muscularity: How female bodybuilders negotiate normative femininity in competitive bodybuilding. Women's Stud Q.

[18] Shilling C, Bunsell T (2009). The female bodybuilder as a gender outlaw. Qual Res Sport Exercise.

[19] Campbell F, Haverda MB, Bartkowski JP (2021). Rival bodies: Negotiating gender and embodiment in women’s bikini and figure competitions. Social Sci.

[20] Van de Ven K, Maher L, Wand H, Memedovic S, Jackson E, Iversen J (2018). Health risk and health seeking behaviours among people who inject performance and image enhancing drugs who access needle syringe programs in Australia. Drug Alcohol Rev.

[21] Kanayama G, Pope HG (2018). History and epidemiology of anabolic androgens in athletes and non-athletes. Mol Cell Endocrinol.

[22] Kanayama G, Kaufman MJ, Pope HG (2018). Public health impact of androgens. Curr Opin Endocrinol Diabetes Obes.

[23] Hartgens F, Kuipers H (2004). Effects of androgenic-anabolic steroids in athletes. Sports Med.

[24] Kanayama G, Brower KJ, Wood RI, Hudson JI, Pope HG (2010). Treatment of anabolic–androgenic steroid dependence: emerging evidence and its implications. Drug Alcohol Depend.

[25] Wood RI (2008). Anabolic–androgenic steroid dependence? Insights from animals and humans. Front Neuroendocrinol.

[26] Sagoe D, Molde H, Andreassen CS, Torsheim T, Pallesen S (2014). The global epidemiology of anabolic-androgenic steroid use: a meta-analysis and meta-regression analysis. Ann Epidemiol.

[27] Abrahin O, Félix Souza NS, de Sousa EC, Santos AM, Bahrke MS (2017). Anabolic–androgenic steroid use among Brazilian women: an exploratory investigation. J Substance Use.

[28] Chadwick KA, Simpson CB, McGarey PO, Estes CM, Nix J, Sulica L (2021). Voice change following testosterone supplementation in women: a multi-institutional case series. J Voice.

[29] Gruber AJ, Pope HG (2000). Psychiatric and medical effects of anabolic-androgenic steroid use in women. Psychother Psychosom.

[30] Scarth M, Jørstad ML, Reierstad A, Klonteig S, Torgersen S, Hullstein IR (2022). Psychopathology among anabolic-androgenic steroid using and non-using female athletes in Norway. J Psychiatr Res.

[31] Chegeni R, Notelaers G, Pallesen S, Sagoe D (2021). Aggression and psychological distress in male and female anabolic-androgenic steroid users: a multigroup latent class analysis. Front Psych.

[32] Ip EJ, Barnett MJ, Tenerowicz MJ, Kim JA, Wei H, Perry PJ (2010). Women and anabolic steroids: an analysis of a dozen users. Clin J Sport Med.

[33] Havnes IA, Jørstad ML, Innerdal I, Bjørnebekk A (2021). Anabolic-androgenic steroid use among women–A qualitative study on experiences of masculinizing, gonadal and sexual effects. Int J Drug Policy.

[34] Zahnow R, McVeigh J, Ferris J, Winstock A (2017). Adverse effects, health service engagement, and service satisfaction among anabolic androgenic steroid users. Contemp Drug Probl.

[35] Havnes IA, Jørstad ML, Wisløff C (2019). Anabolic-androgenic steroid users receiving health-related information; health problems, motivations to quit and treatment desires. Substance Abuse Treatm Prevent Policy.

[36] Piatkowski TM, Hides LM, White KM, Obst PL, Dunn M. Understanding harm reduction perspectives of performance and image enhancing drug consumers and health care providers. Performance Enhancement & Health. 2022:100223.

[37] Piatkowski T, Robertson J, Dunn M. Polysubstance use practices among women using anabolic-androgenic steroids (AAS). Performance Enhancement & Health. 2023:100248.

[38] Gibson K, Hutton F (2021). Women who inject drugs (WWID): Stigma, gender and barriers to needle exchange programmes (NEPs). Contemp Drug Probl.

[39] Grundetjern H (2015). Women's gender performances and cultural heterogeneity in the illegal drug economy. Criminology.

[40] Ettorre E (2004). Revisioning women and drug use: gender sensitivity, embodiment and reducing harm. Int J Drug Policy.

[41] Shirley-Beavan S, Roig A, Burke-Shyne N, Daniels C, Csak R (2020). Women and barriers to harm reduction services: a literature review and initial findings from a qualitative study in Barcelona. Spain Harm Reduction J.

[42] Neuendorf KA (2018). Content analysis and thematic analysis. Advanced research methods for applied psychology.

[43] Poucher ZA, Tamminen KA, Caron JG, Sweet SN (2020). Thinking through and designing qualitative research studies: a focused mapping review of 30 years of qualitative research in sport psychology. Int Rev Sport Exerc Psychol.

[44] Clarke V, Braun V, Hayfield N (2015). Thematic analysis. Qual Psychol Pract Guide Res Methods.

[45] Braun V, Clarke V (2019). Reflecting on reflexive thematic analysis. Qual Res Sport Exercise health.

[46] Braun V, Clarke V, Hayfield N, Terry G. Thematic analysis In: Liamputtong P, editor. Handbook of Research Methods in Health Social Sciences Singapore: Springer. 2018:1–18.

[47] Krane V (2001). We can be athletic and feminine, but do we want to? Challenging hegemonic femininity in women's sport. Quest.

[48] Ainsworth N, N Thrower S, Petróczi A. Fragile femininity, embodiment, and self-managing harm: an interpretative phenomenological study exploring the lived experience of females who use anabolic-androgenic steroids. Qualitative Research in Sport, Exercise and Health. 2022;14(3):363–81.

[49] McGrath SA, Chananie-Hill RA (2009). “Big freaky-looking women”: Normalizing gender transgression through bodybuilding. Sociol Sport J.

[50] Yager Z, McLean S (2020). Muscle building supplement use in Australian adolescent boys: relationships with body image, weight lifting, and sports engagement. BMC Pediatr.

[51] Sagoe D, Andreassen CS, Pallesen S (2014). The aetiology and trajectory of anabolic-androgenic steroid use initiation: a systematic review and synthesis of qualitative research. Substance Abuse Treatm Prevent Policy.

[52] Dunn M, Piatkowski T, Robertson J, Lamon S. Is the use of performance and image enhancing drugs (PIEDs) in women an issue of concern? The findings from a stakeholder consultation Under Review. 10.1016/j.jsams.2023.08.179 37684155

[53] Andreasson J, Henning A (2022). Challenging hegemony through narrative: Centering women’s experiences and establishing a sis-science culture through a women-only doping forum. Commun Sport.

[54] Djordjevic F, Ryan K, Gunn J, Brener L, O’Keefe D, Draper B (2021). Health service utilization and experiences of stigma amongst people who inject drugs in Melbourne. Aust J Viral Hepatitis.

[55] Muncan B, Walters SM, Ezell J, Ompad DC (2020). “They look at us like junkies”: influences of drug use stigma on the healthcare engagement of people who inject drugs in New York City. Harm Reduct J.

[56] Gibbs N, Piatkowski T (2023). The Liver King Lie: Misrepresentation, justification, and public health implications. Int J Drug Policy.

[57] Tighe B, Dunn M, McKay FH, Piatkowski T (2017). Information sought, information shared: exploring performance and image enhancing drug user-facilitated harm reduction information in online forums. Harm Reduct J.

[58] Henning A, Andreasson J (2021). “Yay, another lady starting a log!”: Women’s fitness doping and the gendered space of an online doping forum. Commun Sport.

[59] Iversen J, Page K, Madden A, Maher L. HIV, HCV and health-related harms among women who inject drugs: Implications for prevention and treatment. Journal of acquired immune deficiency syndromes (1999). 2015;69(0 1):S176. 10.1097/QAI.0000000000000659 PMC4505917 25978485

